# Ceruloplasmin overexpression is associated with oncogenic pathways and poorer survival rates in clear‐cell renal cell carcinoma

**DOI:** 10.1002/2211-5463.13283

**Published:** 2021-09-28

**Authors:** Yong Zhang, Zhan Chen, Jian‐Gang Chen, Xin‐Feng Chen, Dong‐Hua Gu, Zhen‐Min Liu, Ya‐Dong Gao, Bing Zheng

**Affiliations:** ^1^ Department of Urology The Second Affiliated Hospital of Nantong University China; ^2^ Department of Medical Research Center The Second Affiliated Hospital of Nantong University China; ^3^ Department of Gastroenterology The Second Affiliated Hospital of Nantong University China

**Keywords:** bioinformatics analysis, biomarker, diagnosis, differentially expressed genes, prognosis, renal cell carcinoma

## Abstract

Clear‐cell renal cell carcinoma (ccRCC) is the most prevalent renal malignancy. The pathogenesis of the disease is currently poorly understood, and the prognosis is poor. Therefore, in this study, we focused on exploring and identifying genes and signal transduction pathways that are closely related to ccRCC. Differentially expressed genes (DEGs) were analyzed using the renal cell oncogene expression profiles GSE100666 and GSE68417. DAVID evaluation of gene ontology (GO) and Kyoto Encyclopedia of Genes and Genomes (KEGG) analyses was used. We constructed a protein–protein interaction (PPI) network of DEGS using Cytoscape software and analyzed the submodules with the CytoHubba plugin. Finally, we performed western blot, immunohistochemistry, and PCR validation by collecting tissues, and also utilized cells for *in vitro* functional analysis of ceruloplasmin (CP). In total, 202 DEGs (52 upregulated and 150 downregulated genes) were identified. Upregulated DEGs are significantly rich in angiogenesis, cell adhesion, and response to hypoxia, whereas downregulated DEGs are involved in intracellular pH regulation, excretion, coagulation, and chloride transmembrane transport. We selected the interactions of the top 20 hub genes provided by the PPI network, all of which are involved in important physiological pathways *in vivo*, such as complement and coagulation cascades. Tissue protein assays demonstrated that renal cancer highly expressed CP, while *in vitro* experiments showed that CP could promote the invasion of renal cancer cells. Our study suggests that ALB, C3, LOX, HRG, CXCR4, GPC3, SLC12A3, CP, and CASR may be involved in the development of ccRCC, and is expected to provide theoretical support for future studies on the diagnosis and targeted therapy of ccRCC.

## Graphical abstract

DEGs in clear cell renal cell carcinoma (ccRCC) were identified and their expression analyzed in ccRCC patient tissues and an RCC cell line. Tissue protein assays demonstrated that renal cancer highly expressed ceruloplasmin (CP), and *in vitro* experiments showed that CP could promote the invasion of renal cancer cells. Overall, CP may promote the poor prognosis of renal cancer.

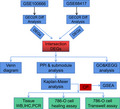

**Abbreviations**
ccRCC: clear‐cell renal cell carcinoma
CP: ceruloplasmin
DEGs: differentially expressed genes
GEO: gene expression omnibus
KEGG: Kyoto Encyclopedia of Genes and Genomes
MF: molecular function
PPI: protein–protein interaction
TCGA: The Cancer Genome Atlas

Renal cell carcinoma (RCC) is a highly prevalent malignancy in the urinary system, second only to bladder cancer, accounting for 2%–3% of adult malignancies [1]. Of these, the most common pathological type of RCC is clear‐cell renal cell carcinoma (ccRCC), which accounts for approximately 85% [2]. Because early symptoms of RCC are not obvious and the patient's tumor has already metastasized at presentation, current medical treatment is not effective for patients with ccRCC metastasis, resulting in a very poor prognosis for ccRCC patients [3, 4]. Therefore, there is an urgent need to find new molecules closely related to the development of ccRCC to improve the current dilemma of poor efficacy.

With the development of gene sequencing technology, tumor markers have been widely used for clinical diagnosis and treatment of tumors, prognosis, and efficacy assessment [5]. Analysis of the molecular mechanisms of tumor pathogenesis will help to develop more effective preventive and therapeutic strategies. In recent years, microarrays have achieved some success in identifying epigenetics related to neoplastic diseases, which has prompted an increasing application to the study of malignant tumor diseases. However, performing independent microarray analysis in individual studies is incidental and not representative, making it difficult to identify reliable key genes and pathways. To identify novel differentially expressed genes (DEGs) as effective biomarkers for diagnosis and prognosis of ccRCC, we combined two microarray mRNA microarray datasets from the Gene Expression Omnibus (GEO) database. To determine the reliability of screening results, the Cancer Genome Atlas (TCGA) database was also used to assist validation and seek gene molecules most associated with ccRCC progression.

In this study, we selected differential genes by GEO2R and then constructed a protein–protein interaction (PPI) network based on DEGs to identify key genes and related signaling pathways that may be involved in RCC development. We found some significant genes, especially CP. CP is an α2‐glycoprotein that is mainly produced by hepatocytes. It is the main ferroxidase in the human body and is important to regulate both systemic and intracellular iron levels [6]. It has been found that CP is also considered as an important inflammatory response protein, through interleukin‐1 and interleukin‐6 stimulation, enabling it to scavenge oxygen free radicals at the site of inflammation [7, 8]. On the other hand, studies have shown that CP also plays an important role in angiogenesis [9], while many reports have shown that CP content is high in the serum of patients with malignant tumors [10, 11] and in tumor tissues including the kidney [12]. The results of this study are expected to provide new theoretical support to identify novel targeted actionable genes and possible clinical biomarkers for clear‐cell renal carcinoma.

## Materials and methods

### RNA‐seq and microarray data

The mRNA expression profiles of datasets GSE100666 and GSE68417 were downloaded from the GEO database, where GSE100666 consisted of three renal cell carcinoma samples and three normal tissue samples, and the raw data processed by Phalanx Human OneArray Ver. 6 Release 1. GSE68417 consist of 29 RCC samples and 20 non‐cancerous samples, raw data processed by Affymetrix Human Gene 1.0 ST Array. Transcriptional expression data from renal cancer patients were obtained in the TCGA database. Including: Case: Primary Site: kidney, Program: TCGA, Project: TCGA‐KIRC. Files: Data Category: transcriptome profiling, Data Type: Gene Expression Quantification, Experimental Strategy: RNA‐Seq, Workflow Type: HTSeq‐FPKM. Eventually, we screened a total of 611 sequencing files.

### Identified genes of differential expression

We screened DEGs between RCC and noncancerous samples by GEO2R (http://www.ncbi.nlm.nih.gov/geo/geo2r), an analytical tool of the GEO database. Consistent with the criteria of previous investigators (6, 7), we defined log2FC < −2 (downregulated genes) or log2FC > 2 (upregulated genes) as DEGs. In the TCGA database, we assessed whether the two groups were statistically different by Wilcoxon test using the gene expression after normalization of tumor tissue and normal tissue. *P* < 0.05 was considered statistically significant

### Gene ontology and KEGG pathway analysis of DEGs

For functional enrichment analysis of the screened DEGs, we performed the analysis by the online program DAVID database to annotate different genes, allowing us to better understand the biological functions of genes (8). *P*‐values < 0.05 were used to distinguish significantly enriched genes.

### PPI network analysis

To assess the interaction of DEGs, we submitted DEGs to the interacting gene/protein (STRING) database (https://string‐db.org/), network (PPI), and to construct predicted protein‐protein interactions (interaction score > 0.4), the constructed PPI network was displayed with Cytoscape software. In this study, the degree values are used to evaluate the nodes in the network. CytoHubba is a plugin for Cytoscape for predicting key nodes and subnets in a network. The top 20 ranked genes were selected and identified as the central genes for degree ranking.

### Kaplan–Meier survival analysis of Hub Gene

The impact of screened central genes on the survival of patients with kidney cancer was analyzed by using the online tool Kaplan–Meier plotter (KM plotter), including mRNA, RNA‐seq, pan‐cancer, and kidney renal clear‐cell carcinoma. Renal cell carcinoma patients were divided into two groups (high and low expression) based on auto select best cutoff parameter expression levels of specific genes. OS was calculated for both groups and plotted, and hazard ratios (HR) and log‐rank P were calculated with 95% confidence intervals (CI).

### Hub genes validation

GEPIA (http://gepia.cancer‐pku.cn/index.html), the RNA‐sequencing expression data of tumor tissue samples, and normal tissue samples were obtained from the TCGA database and the GTEx database, respectively (10), and it can be directly used for tumor/normal tissue differential expression analysis. In this study, GEPIA was used to validate the expression differences of the hub genes. We defined this difference as statistically significant (*P* < 0.05).

### Gene set enrichment analysis

According to the mRNA expression level of CP, 539 clear renal cell carcinoma samples from the TCGA database were divided into high and low expression groups using the median value as the cutoff point, and Gene set enrichment analysis (GSEA) software was used to analyze the enrichment results. *P* < 0.05 and FDR < 25% were selected as cutoff criteria [13].

### Tissue samples and cell culture

From August 2017 to December 2017, a total of four pairs of ccRCC tissues and adjacent normal tissues were obtained from the Second Affiliated Hospital of Nantong University. The experiments were performed with the understanding and written consent of each subject, and the methods of this study complied with the standards laid down in the Declaration of Helsinki. The study was approved by the Human Research Ethics Committee of the Second Affiliated Hospital of Nantong University, and written informed consent was obtained from the patients. Clear renal cell carcinoma cell line 786‐O was purchased from Shanghai Institute of Cellular Biochemistry, Chinese Academy of Sciences (Shanghai, China). The cells were cultured in 1640 medium with 10% fetal bovine serum at 37 °C, 5% carbon dioxide, harvested when the cells were in the logarithmic growth phase, and used for subsequent experiments.

### Transient transfection assay

SiRNA for CP (si‐CP) and negative control (si‐NC) were chemically synthesized by GenePharma (Shanghai, China). The working concentration of Si‐CP and Si‐NC was 40 nm. In this study, Lipofectamine® 2000 (Invitrogen, Waltham, MA, USA) and siRNA were diluted with 250 μL of OPTI‐MEM, respectively, allowed to stand for 5 min, mixed well and allowed to stand for 30 min again, and finally added to a six‐well plate to incubate with cells for 6 h, changed to 1640 medium containing 10% fetal bovine serum for another 48 h before use in the experiment.

### qRT‐PCR

In this experiment, total RNA was extracted from cells and tissues by using TRIzol reagent (Invitrogen, Carlsbad, CA, USA). RT‐PCR was performed on RNA samples using TaKaRa one‐step RT‐PCR kit (Takara, Shiga, Japan). For real‐time PCR, cDNA was analyzed in triplicate using SYBR Green (Takara). Relative mRNA concentrations were determined from 2^−ΔΔCt^.

### Immunohistochemistry

Paraffin‐embedded sections were deparaffinized and hydrated. Slides were placed in citrate buffer (pH 6.0) and heated for 3 min at high pressure. Incubate with 0.3% H2O2 solution at room temperature for 15 min, rinse with PBS for 3 × 3 min, then block with goat serum for 30 min, then incubate primary antibody anti‐CP (1 : 200, Proteintech, Wuhan, China) with sections overnight at 4 °C and wash with PBS for 3 × 3 min, then incubate secondary antibody (1 : 5000, Proteintech, Wuhan, China) and sections at room temperature for 30 min, then incubate with peroxidase‐antiperoxidase complex at room temperature for 30 min, rinse with PBS for 3 × 3 min, then stain with DAB, finally counterstain nuclei with hematoxylin, mount with neutral gum and observe sections under microscope.

### Western blotting

SI‐NC cells, SI‐CP cells, and kidney cancer patient tissue samples were collected and lysed with RIPA lysis buffer (Beyotime, Nanjing, China) on ice and added to a final concentration of 1 mm PMSF (Beyotime, Nanjing, China) for 30 min. The lysates were centrifuged at 12 000 ***g*** for 15 min at 4 °C, and the supernatants were collected. Protein concentration was determined using the Bradford protein assay kit (ThermoScience, MA, USA). Approximately 40 μg of protein was loaded by SDS/PAGE gel electrophoresis, and then, the proteins were transferred to PVDF membranes. After blocking with 5% skimmed milk in TBST buffer for 2 h, PVDF membranes were incubated with anti‐CP (1 : 1000, Wuhan, Proteintech) and GAPDH antibodies (1 : 5000, Wuhan, Proteintech) overnight at 4 °C. After washing the PVDF membrane with PBST for 10 min*3, the secondary antibody conjugated with horseradish peroxidase (1 : 5000, Proteintech, Wuhan, China) was incubated at room temperature for 1 h. After washing the PVDF membrane with PBST for 10 min*3, the bands were detected using a chemiluminescent substrate ECL kit (Merck Millipore, Danvers, MA, USA).

### Invasion assay

Diluted matrigel (diluted 1 : 6 with serum‐free medium) was added to the cell culture chamber, and after Matrigel solidification, 100 μL of SI‐CP and SI‐NC cells at a concentration of about 10^6^/mL was added to the upper chamber, and 500 μL of 20% fetal bovine serum containing medium was placed in the lower chamber and routinely cultured for 24 h before removing the cells on the upper side of the chamber with a cotton swab, and the cells under the filter membrane were fixed with 4% paraformaldehyde for 10 min and stained with crystal violet for 10 min. Three randomly selected areas under the microscope were used for counting the stained cells. The number of cells in each region was used as a statistical result.

### Wound healing assay

Cells 48 h after siRNA transfection were cultured in 6‐well plates containing serum medium, and when the cell density reached 80%, the cells were washed three times with sterile PBS, and the bottom of the six‐well plate was scraped with a 200‐μL pipette tip, the debris was removed by PBS, and the cells were cultured in serum‐free medium for 12 h, cell migration was analyzed in three randomly different microscopic fields and the percentage of wound healing was calculated. Images were captured on a microscope at 0 and 12 h.

### Statistical analysis

graphpadprism5 (San Diego, CA, USA) and spss 22.0 software (Chicago, IL, USA) were used for all statistical analyses. Quantitative data were expressed as mean ± SD. Significant differences in quantitative data were compared by unpaired *t*‐test. *P*‐values < 0.05 were considered statistically significant. Statistical significance was determined as #*P* < 0.05, ##*P* < 0.01, and ###*P* < 0.001.

## Results

### Screening of DEGs

**Fig. 1:**
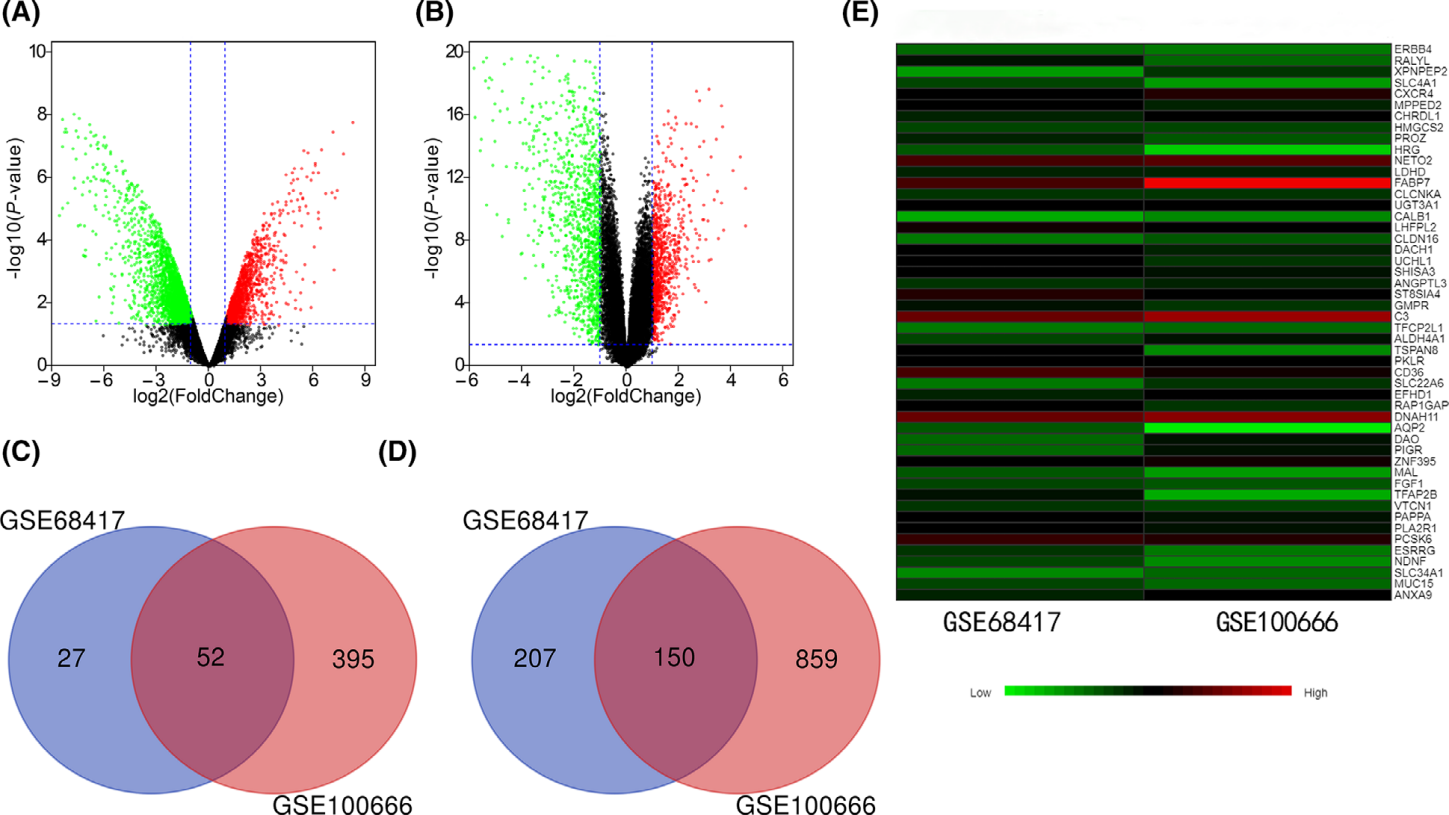
DEGs volcano, Venn, and heat maps. The volcano graph (A) is the result of the GSE100666 data set, and the volcano graph (B) represents the result of the GSE68417 data set. The abscissa is log2FC, and the ordinate is ‐log10 (*P* value). The red and green spots represent upregulated and downregulated DEGs, respectively. The Venn diagram (C) represents the common upregulation of DEGs in the two datasets, and the Venn diagram (D) represents the common downregulation of DEGs in the two datasets. Heat map (E) represents the top 50 of DEGs where the two data sets intersect.

According to the screening criteria in the Materials and Methods section, 1455 and 438 DEGs were obtained from the GSE100666 and GSE68417 datasets, respectively (Fig. 1A,B). The two data sets were intersected to have a total of 205 genes, of which three genes had opposite expression levels in the two data sets and were removed in this study, leaving 202 genes for subsequent analysis. The results showed that 52 genes were upregulated and 150 genes were downregulated in tumor tissue samples compared with non‐cancerous samples (Fig. 1C,D). The heatmap shows the top 50 DEGs with the most pronounced differences in common between the two datasets (Fig. 1E). Green indicates downregulation, and red indicates upregulation.

### Analysis of upregulated and downregulated differential genes in GO and KEGG pathways

**Fig. 2:**
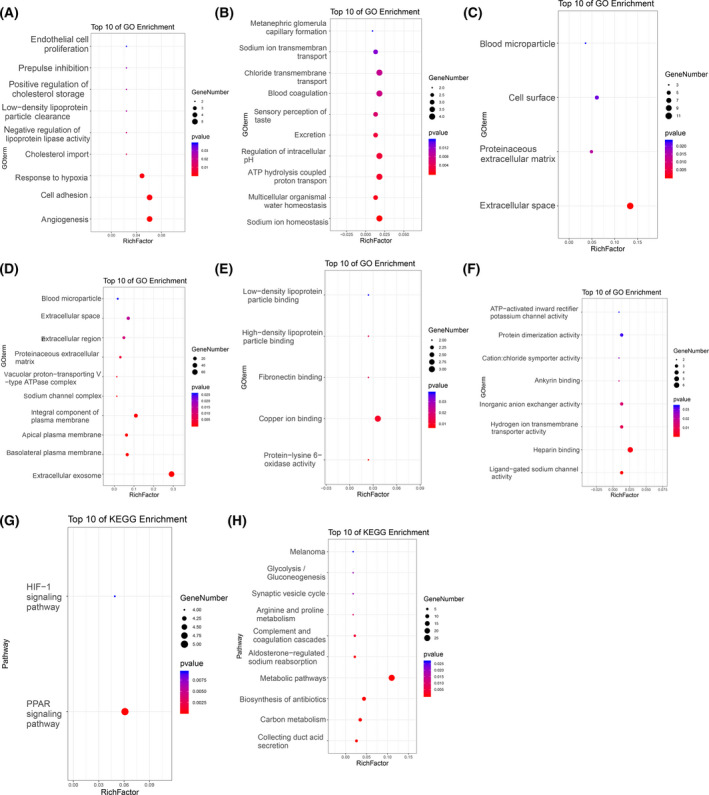
GO and KEGG analysis of DEGs. (A), Biological process termed for upregulated DEGs. (B), Biological process terms for downregulated DEGs. (C), Cellular component terms for upregulated DEGs. (D), Cellular component terms for downregulated DEGs. (E), Molecular function terms for upregulated DEGs. (F), Molecular function terms for downregulated DEGs. (G), KEGG pathway of upregulated DEGs. (H), KEGG pathway of downregulated DEGs.

GO and Kyoto Encyclopedia of Genes and Genomes (KEGG) enrichment analysis of upregulated and downregulated DEG using David tool. As far as biological processes (BP) are concerned, the increased DEGs is significantly enriched angiogenesis, cell adhesion, and response to hypoxia (Fig. 2A). Downregulated DEGs were involved in sodium ion homeostasis, multicellular organismal water homeostasis, ATP hydrolysis coupled proton transport, regulation of intracellular pH, excretion, blood coagulation, and chloride transmembrane transport (Fig. 2B). The analysis of cell composition (CC) showed that the upregulated DEGS was significantly enriched in extracellular space, proteinaceous extracellular matrix, cell surface, and blood microparticle (Fig. 2C), while the downregulated DEGs were significantly enriched in extracellular exosome, absolutely plasma membrane, apical plasma membrane, integral component of plasma membrane, extracellular region, and extracellular space (Fig. 2D). In terms of molecular function (MF), upregulated DEGs were significantly enriched in copper ion binding, protein‐lysine 6‐oxidase activity, fibronectin binding, and high‐density lipoprotein particle binding (Fig. 2E), while the downregulated DEGs were significantly enriched in heparin binding, ligand‐gated sodium channel activity, hydrogen ion transmembrane transporter activity, inorganic anion exchanger activity, and protein dimerization activity (Fig. 2F). KEGG analysis showed that the upregulated DEGs were strikingly enriched in PPAR signaling pathway and HIF‐1 signaling pathway (Fig. 2G), while the downregulated DEGs were significantly enriched in collecting duct acid secretion, carbon metabolism, biosynthesis of antibiotics, and metabolic pathways (Fig. 2H).

### PPI network construction and related signaling pathways for hub gene enrichment

**Fig. 3:**
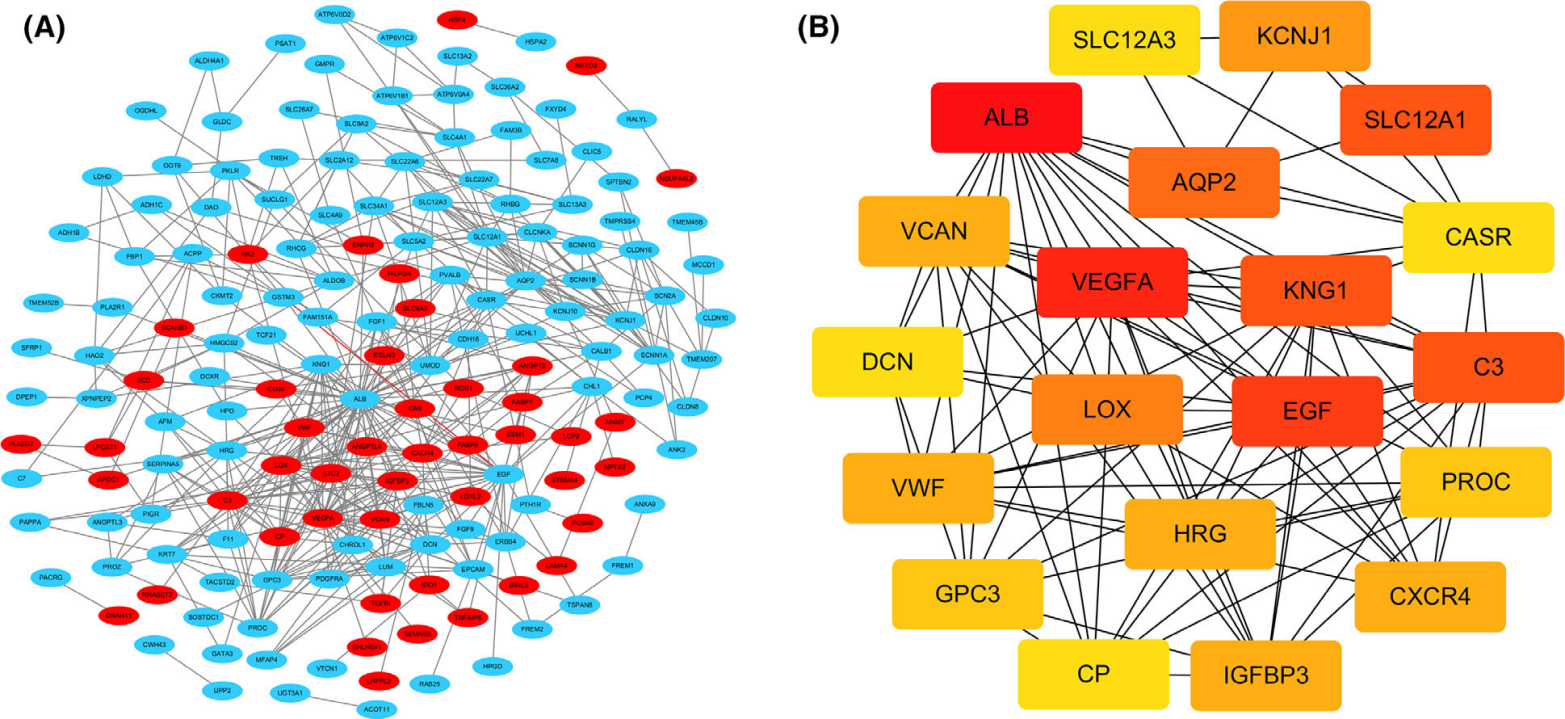
PPI network of DEGs and submodule analysis. (A) Build all DEG into PPI network through Cytoscape software. Red represents upregulated genes, and blue represents downregulated genes. (B) The PPI network of the top 20 hub genes.

The 202 DEGs obtained above were analyzed by PPI network online, and a PPI network with 202 nodes and 482 edges was constructed. The PPI enriched p value < 1.0 × 10^−16^. The network contains 52 upregulated genes and 150 downregulated genes (Fig. 3A). In this study, the cytoHubba plugin was used to select the top 20 genes with the highest correlation from the PPI network as hub genes. The top 20 hub genes are as follows: ALB, VEGFA, EGF, KNG1, C3, SLC12A1, AQP2, LOX, KCNJ1, VWF, VCAN, HRG, IGFBP3, CXCR4, PROC, GPC3, SLC12A3, DCN, CP, and CASR (Fig. 3B). The GO function and KEGG pathway enrichment of the above core genes were analyzed by DAVID, so that we can have a more comprehensive and in‐depth understanding of these core genes. Table 1 shows the TOP5 gene ontology categories. In terms of biological process (BP), hub gene is mainly enriched in the negative regulation of vascular endothelial growth factor signal pathway, negative regulation of blood coagulation, positive regulation of focal adhesion assembly, platelet activation, and negative regulation of apoptosis process. In cell component (CC), hub genes were mainly enriched in extracellular exosome, blood microparticle, proteinaceous extracellular matrix, extracellular space, and apical plasma membrane. In addition, in MF, the hub genes were mainly enriched in cation: chloride symporter activity, fibronectin binding, collagen binding, heparan sulfate proteoglycan binding, and cysteine‐type endopeptidase inhibitor activity (Table 1). Table 2 reveals the most significant KEGG pathway among the top 20 central genes. These genes were enriched in Complement and coagulation cascades (Table 2).

**Table 1:** Gene ontology enrichment analysis of top 20 hub genes.

Category	Term	Count	*P* value
GOTERM_BP_DIRECT	GO:1900747˜negative regulation of vascular endothelial growth factor signaling pathway	2	0.007687913
GOTERM_BP_DIRECT	GO:0030195˜negative regulation of blood coagulation	2	0.009218762
GOTERM_BP_DIRECT	GO:0051894˜positive regulation of focal adhesion assembly	2	0.022896079
GOTERM_BP_DIRECT	GO:0030168˜platelet activation	2	0.037883252
GOTERM_BP_DIRECT	GO:0043066˜negative regulation of apoptotic process	3	0.039124532
GOTERM_CC_DIRECT	GO:0070062˜extracellular exosome	13	2.34E‐06
GOTERM_CC_DIRECT	GO:0072562˜blood microparticle	5	2.83E‐06
GOTERM_CC_DIRECT	GO:0005578˜proteinaceous extracellular matrix	5	5.98E‐05
GOTERM_CC_DIRECT	GO:0005615˜extracellular space	8	6.18E‐05
GOTERM_CC_DIRECT	GO:0016324˜apical plasma membrane	3	0.081389039
GOTERM_MF_DIRECT	GO:0015377˜cation:chloride symporter activity	2	0.004898581
GOTERM_MF_DIRECT	GO:0001968˜fibronectin binding	2	0.008151664
GOTERM_MF_DIRECT	GO:0005518˜collagen binding	2	0.011394669
GOTERM_MF_DIRECT	GO:0043395˜heparan sulfate proteoglycan binding	2	0.017850569
GOTERM_MF_DIRECT	GO:0004869˜cysteine‐type endopeptidase inhibitor activity	2	0.032230616

**Table 2:** KEGG pathway enrichment analyses of top 20 hub genes.

Category	Term	Count	*P* value
KEGG_PATHWAY	cfa04610:Complement and coagulation cascades	4	2.72E‐04

### The Kaplan–Meier survival analysis

**Fig. 4:**
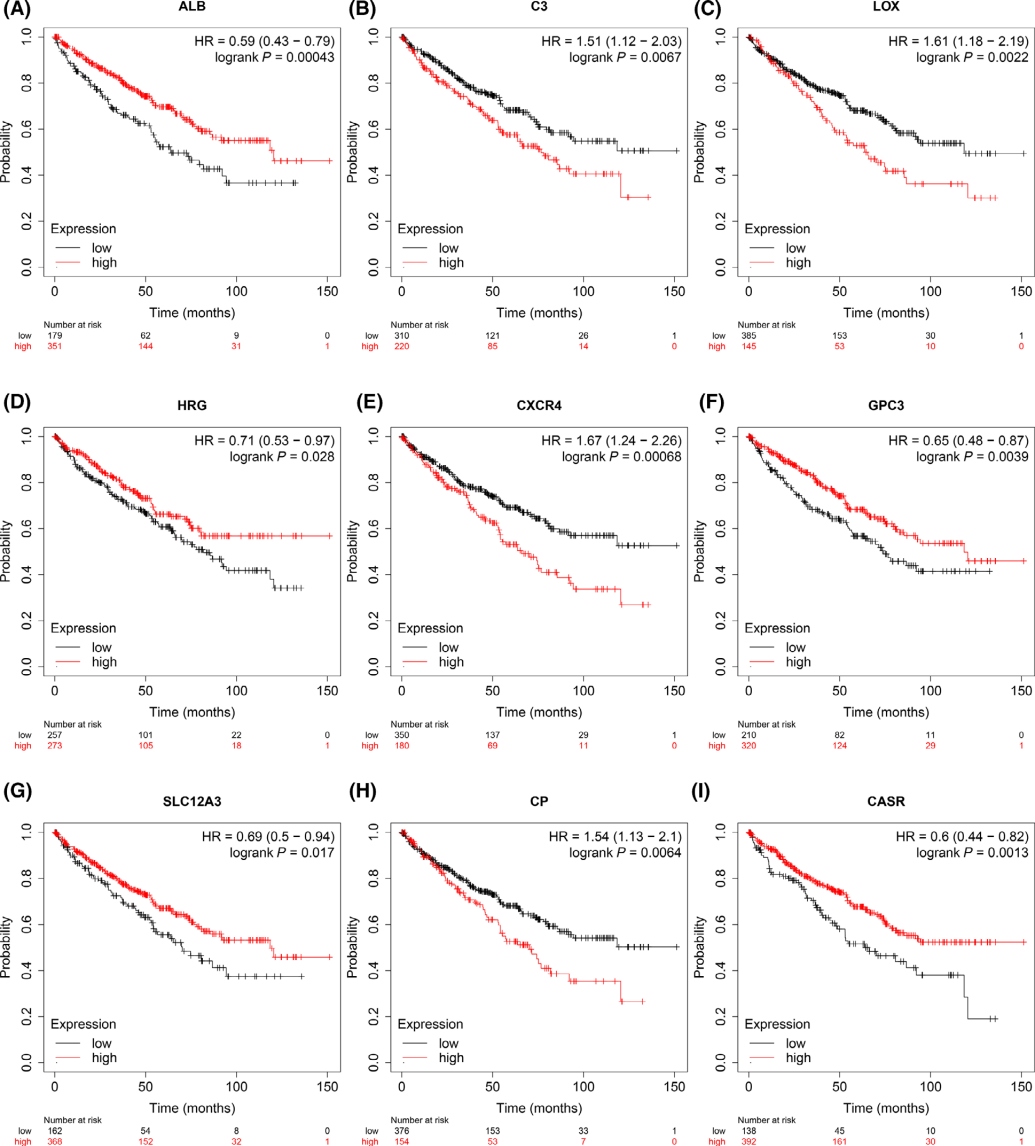
Prognostic estimation of 9 hub genes by Kaplan–Meier analysis. (A) ALB, (B) C3, (C) LOX, (D) HRG, (E) CXCR4, (F) GPC3, (G) SLC12A3, (H) CP, (I) CASR. *P* < 0.05 was as statistically significant.

The prognostic value of the above 20 hub genes was analyzed online by Kaplan–Meier tool. Depending on expression levels, only 9 genes showed their potential in predicting survival, and the remaining genes failed to show significant differences based on prognosis. As shown in Fig. 4, ccRCC patients with high expression of C3 (HR1.51, 95% CI: 1.12–2.03, *P* = 0.0067), LOX (HR1.61, 95% CI: 1.18–2.19, *P* = 0.0022), CXCR4 (HR1.67, 95% CI: 1.24–2.26, *P* = 0.00068), and CP (HR1.54, 95% CI: 1.13–2.1, *P* = 0.0064) usually have a lower overall survival rate. The survival rate of patients with a lower expression of ALB (HR 0.59, 95% CI: 0.43–0.79, *P* = 0.00043), HRG (HR 0.71, 95% CI: 0.53–0.97, *P* = 0.028), GPC3 (HR 0.65, 95% CI: 0.48–0.87, *P* = 0.0039), SLC12A3 (HR 0.69, 95% CI: 0.5–0.94, *P* = 0.017), and CASR (HR 0.6, 95% CI: 0.44–0.82, *P* = 0.0013) tend to be shorter compared with patients over‐expressing those genes (Fig. 4A–I).

### Hub genes are differential expressed between tumoral a nontumoral adjacent tissues in ccRCC

**Fig. 5:**
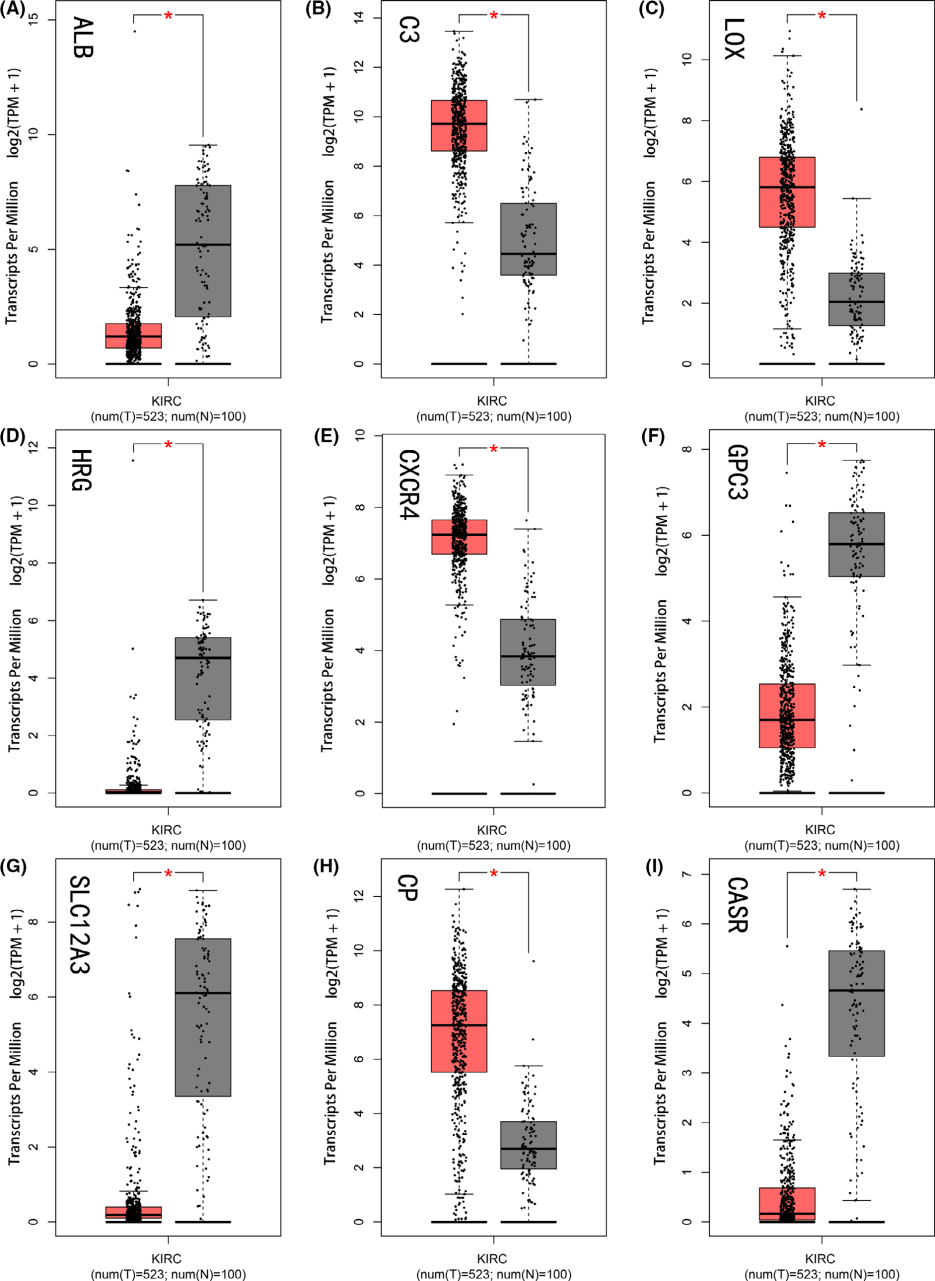
The expression of 9 HUB genes in tumor tissues and normal tissues in GEPIA. The red and gray boxes represent cancer and normal tissue, respectively. (A) ALB, (B) C3, (C) LOX, (D) HRG, (E) CXCR4, (F) GPC3, (G) SLC12A3, (H) CP, (I) CASR. Error bars represent standard deviation. *P < 0.05 was as statistically significant.

In order to increase the reliability of the results, we used the online tool GEPIA, which has data from the TCGA database and the GTEx database to assess the differential expression of the above 9 hub genes again, and the results showed perfect agreement with the GEO dataset (Fig. 5A–I).

### The expression level of CP in ccRCC correlates with TNM stage and histological grade

**Fig. 6:**
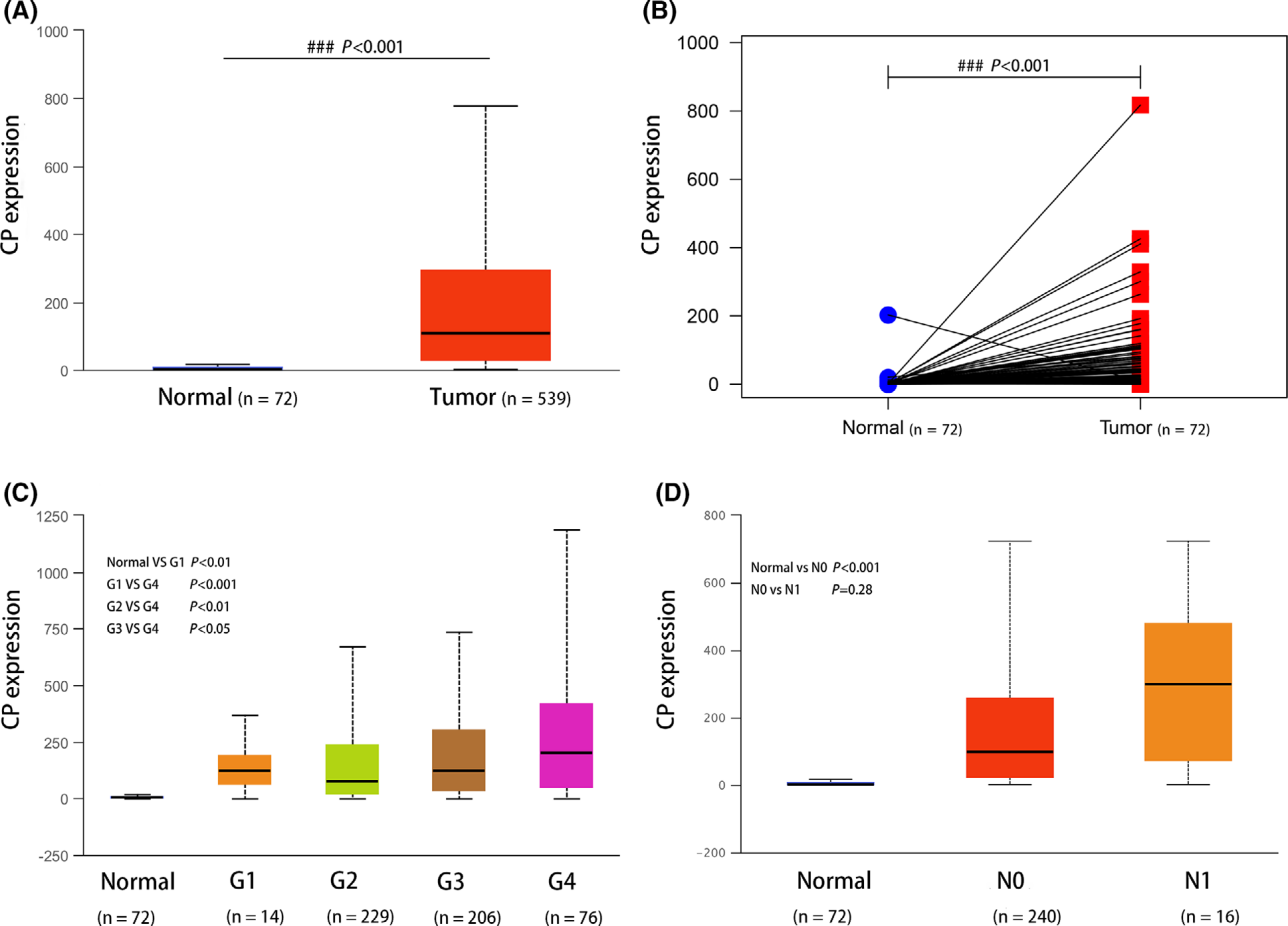
Overexpression of CP in ccRCC was correlated with lymph node metastasis stage and histological grade in the TCGA database. The mRNA expression level of CP in ccRCC tissues and normal renal tissues (A, B). Analysis of CP expression in ccRCC at different histological grades (C) and nodal metastasis status (D). Error bars represent standard deviation. *P* < 0.05 was as statistically significant.

Analysis of 539 ccRCC cases in the TCGA database indicated that the expression difference of CP was significantly statistically significant with clinical prognosis among the above 9 hub genes. Therefore, this study focused on analyzing the biological significance of CP. The expression of CP was significantly higher in the tumor group compared with the control group, and the results were exactly the same by paired comparison of samples from the same patient (Fig. 6A,B). As the pathological grade of the tumor increased and the number of lymph node metastases increased, the expression of CP tended to increase (Fig. 6C,D). These results suggest that CP may have a promoting effect on the progression and metastasis of ccRCC, which is also consistent with the poor survival of patients with high CP expression.

### Diverse oncogenic and tumor progression signaling pathways were enriched in renal tumors over‐expressing CP

**Fig. 7:**
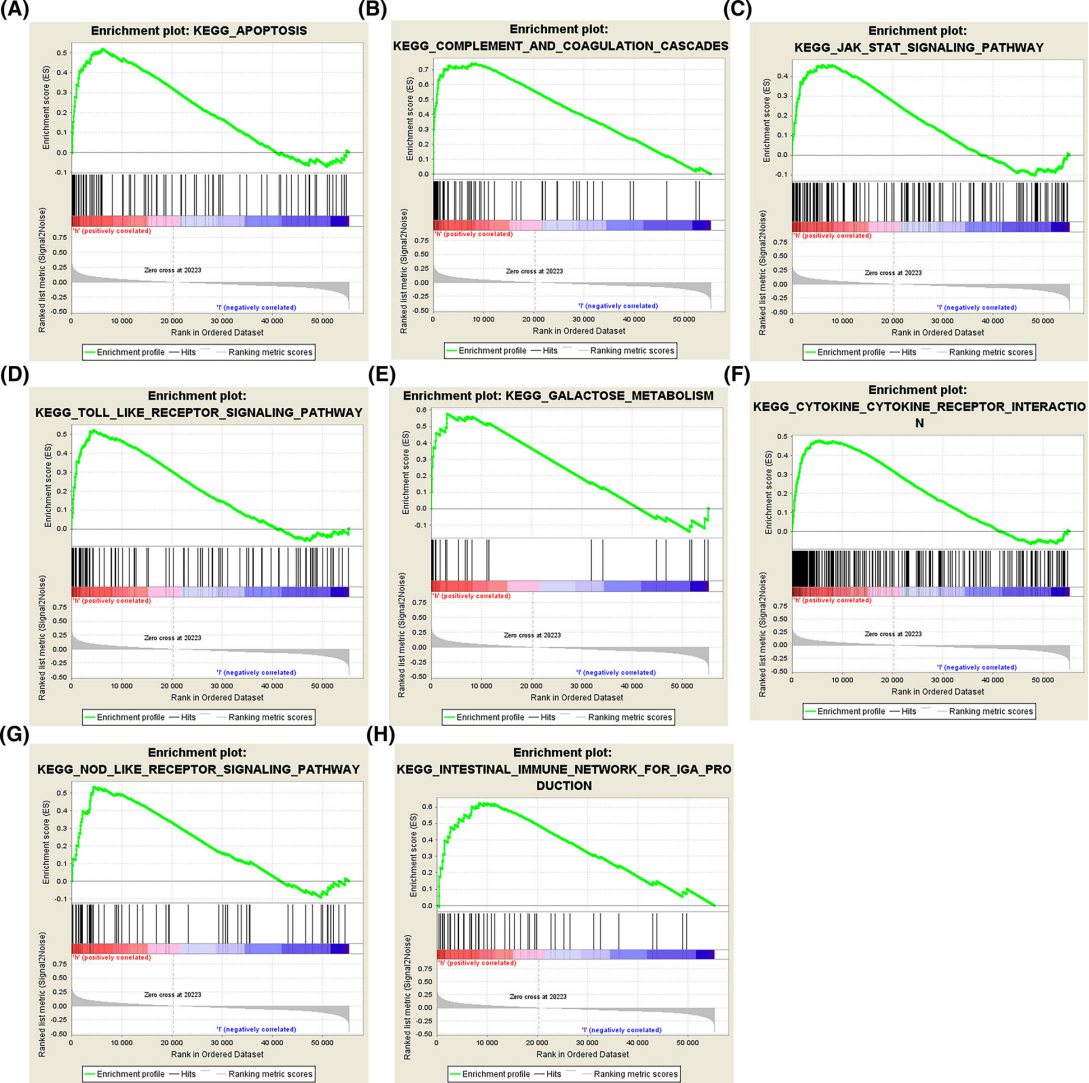
GSEA of CP. Eight representative functional gene sets enriched in ccRCC with highly expressed CP are listed.

To gain more insight into the mechanisms underlying CP in renal cancer progression, GSEA was used to KEGG pathways. According to the previous evaluation criteria, the results showed that highly expressed CP was significantly enriched in *CYTOKINE CYTOKINE RECEPTOR INTERACTION, APOPTOSIS, JAK STAT SIGNALING PATHWAY, COMPLEMENT AND COAGULATION CASCADES*. Other gene sets of important pathways for ccRCC progression and metastasis have also been associated with CP expression (Fig. 7A–H). The details are reported in Table 3.

**Table 3:** Relative pathways associated with the expression of CP. NES, normalized enrichment score; NOM, nominal; FDR, false discovery rate. Gene sets with NOM *P* value < 0.05 and FDR q value < 0.25 are considered as statistical significance.

NAME	ES	NES	NOM *P*‐val	FDR q‐val
KEGG_CYTOKINE_CYTOKINE_RECEPTOR_INTERACTION	0.479410	2.0186532	0.00202839	0.09721641
KEGG_COMPLEMENT_AND_COAGULATION_CASCADES	0.742838	1.9244971	0.00195312	0.10685002
KEGG_TOLL_LIKE_RECEPTOR_SIGNALING_PATHWAY	0.521514	1.9017235	0.01369863	0.10990089
KEGG_APOPTOSIS	0.521048	1.8951145	0.01425661	0.10094964
KEGG_JAK_STAT_SIGNALING_PATHWAY	0.457760	1.8943477	0.00598802	0.09010714
KEGG_INTESTINAL_IMMUNE_NETWORK_FOR_IGA_PRODUCTION	0.623336	1.8751265	0.02376237	0.08605036
KEGG_GALACTOSE_METABOLISM	0.577687	1.8690385	0.00814664	0.08225632
KEGG_NOD_LIKE_RECEPTOR_SIGNALING_PATHWAY	0.536916	1.8579752	0.02008032	0.08112565

### CP is upregulated in ccRCC

**Fig. 8:**
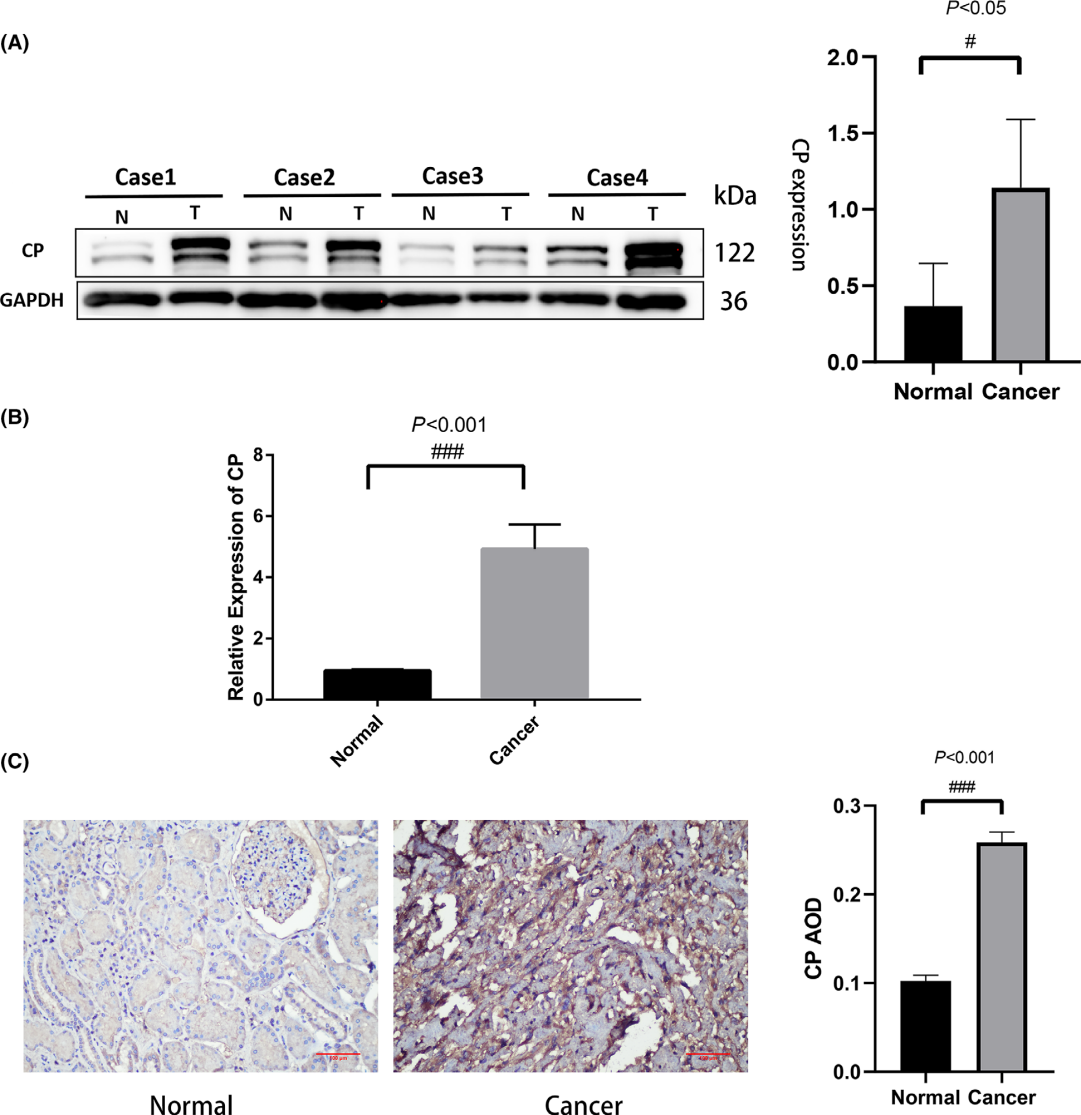
Expression of CP in renal cell carcinoma and adjacent normal tissues. (A) Western blots showing CP expression in renal cell carcinoma and adjacent normal tissues, *n* = 4. (B) Quantitative real‐time RT‐PCR showing CP mRNA expression, *n* = 3. (C) CP immunohistochemical staining for CP in renal cell carcinoma and adjacent normal tissues, *n* = 3. Significant differences in quantitative data were compared by unpaired *t*‐test. Error bars represent standard deviation. Scale bar 100 μm, *P* < 0.05 was as statistically significant.

In order to understand the expression level of CP in ccRCC tissues, we detected the expression level of CP in four pairs of human ccRCC tissues and adjacent non‐tumor tissues using western blotting, and the results showed that the expression level of CP in ccRCC was significantly higher than that in the corresponding adjacent noncancerous tissues (Fig. 8A). In addition, qRT‐PCR was used to detect the mRNA expression of CP in ccRCC and adjacent noncancerous tissues, and the results showed that the mRNA level in ccRCC was significantly higher than that in adjacent non‐cancerous tissues (Fig. 8B). The expression of CP in ccRCC was detected using the ICH method, and CP was markedly upregulated in ccRCC tissues compared with adjacent noncancerous tissues (Fig. 8C). The above experimental results agree well with the results in the database, so we conclude that CP is a potential tumor promoting factor in ccRCC.

### CP promotes the invasion ability of ccRCC cells

**Fig. 9:**
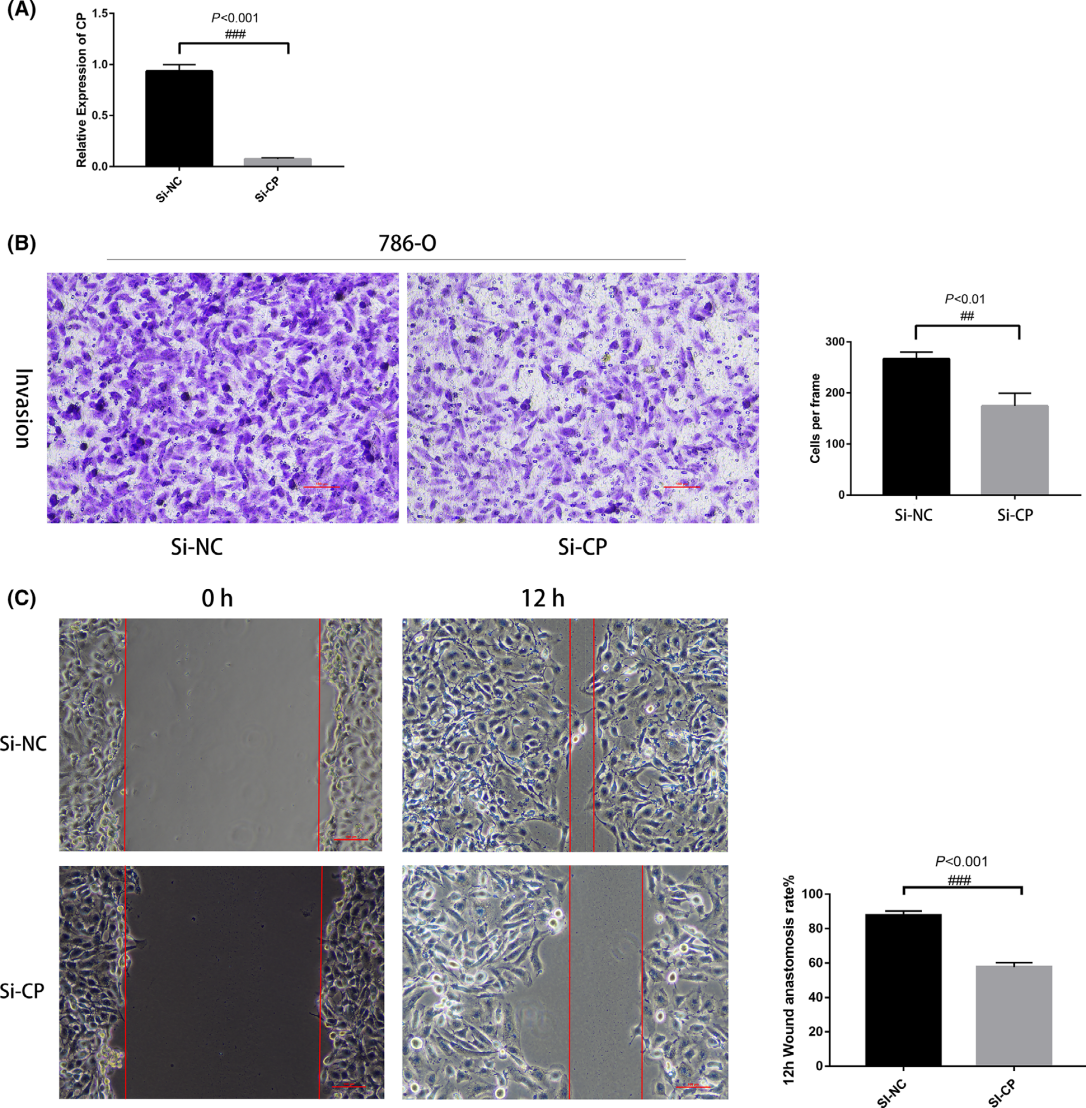
Biological function of CP in 786‐O cell lines. (A) qRT‐PCR showing SI‐CP mRNA expression, *n* = 3. (B) Transwell assay of CP, *n* = 3. (C) Wound healing assay, *n* = 3. Significant differences in quantitative data were compared by unpaired *t*‐test. Error bars represent standard deviation. Scale bar 100 μm, *P* < 0.05 was as statistically significant.

786‐O cells were transfected with SI‐CP and SI‐NC, and 48 h after transfection, qRT‐PCR results showed a significant decrease in CP expression in the SI‐CP group compared with the control group (Fig. 9A). Transwell cell assay was used to examine whether CP had an effect on the invasive ability of ccRCC. The wound healing assay was used to verify whether CP affected the migration ability of ccRCC. The results of cell experiments showed that the in vitro invasive ability of renal cancer cells in the Si‐CP group was significantly lower than that of SI‐NC cells in the control group (Fig. 9B,C). Collectively, our results indicate that CP is involved in the invasion process of ccRCC.

## Discussion

Clear‐cell renal cell carcinoma is still the most common pathological type of renal cell carcinoma. The development and progression of ccRCC is a complex biological process consisting of multifactorial and multigenic interactions and influences through multiple steps and stages. Studies on the molecular mechanisms of ccRCC development are helpful for the development of targeted drugs to guide clinical diagnosis and treatment. In recent years, due to the rapid development of microarray technology and high‐throughput sequencing, it has become a reality to comprehensively study the human genome, and they have now been widely used to explore potential molecular targets for renal cell carcinoma [14].

In this study, GEO2R was used to analyze the differences in gene expression profiles between the GSE100666 and GSE68417 datasets, and a total of 52 upregulated genes and 150 downregulated genes were found. Then, functional and pathway enrichment analysis was performed to discover the potential biological functions of DEGs. The STRING database and Cytoscape were further used to screen the top 20 central genes clearly associated with ccRCC. Because survival analysis, pathological grade, and lymphatic metastasis showed that CP had good diagnostic and prognostic value in these hub genes, CP was selected for further validation, and we used GSEA for gene enrichment analysis, which was finally validated by cell experiments as well as tissue samples. We analyzed the differential genes and the top 20 hub genes separately by GO analysis tool and found that their biological processes were mainly focused on angiogenesis and cell adhesion. According to previous reports, angiogenesis plays an important role in the development of renal cancer, and Goka *et al*. found that by inhibiting VEGF production in ccRCC cells, new angiogenesis can be prevented, which in turn achieves the effect of targeted therapy for renal cancer [15]. More importantly, the NCCN and EAU have used drugs that target inhibition of angiogenesis (sorafenib, sunitinib, etc.) as a first‐line treatment for metastatic renal cell carcinoma [16, 17]. It is known that CD44 antigen is a cell surface glycoprotein involved in cell adhesion and migration, and Mikami *et al*. found that high expression of CD44 is closely related to the metastasis of renal cancer cells, the occurrence of sunitinib resistance, and the poor prognosis of ccRCC [18]. A recent study reported that molecules associated with cell adhesion and extracellular matrix were significantly disturbed in ccRCC and correlated with the survival of renal cancer patients [19].

The results of GSEA analysis showed that high expression of CP was associated with significant enrichment of Complement and coagulation cascades, Toll‐like receptor signaling pathway, apoptosis, and *JAK STAT* signaling pathway. Most of the complement system is secreted by the liver and enters the blood circulation. Some studies have shown that partial complement is involved in the pathological process of renal diseases [20]. Complement cascade and hyper‐coagulation are mutually induced processes in a vicious cycle, and they can promote the tumorigenic phenotype of immune cells and protect tumor cells from immune attack, ultimately facilitating tumor development, progression, and metastasis formation [21]. More evidence also supports the involvement of the complement pathway in cancer [22]. HeeJung found that inhibition of JAK/STAT3 signaling transduction can lead to apoptosis of renal clear‐cell carcinoma, which in turn inhibits tumor progression [23].

According to the survival analysis, 9 DEGs among the above 20 hub genes were closely related to shorter survival time of ccRCC patients, including ALB, C3, LOX, HRG, CXCR4, GPC3, SLC12A3, CP, and CASR. It has been shown that patients with high Alb expression have better progression‐free survival (PFS) and overall survival (OS) than those with low Alb [24]. It has been found that in ccRCC, the expression of C3 is higher than that of C3 in adjacent normal tissues, suggesting that C3 has the potential to interfere with the progression of ccRCC [25]. Similar findings have been made in liver cancer, in which hepatic stellate cells can promote T‐cell apoptosis and reduce their proliferation through the C3 pathway, inhibit the maturation of dendritic cells (DCs), and then promote the development of hepatocellular carcinoma *in vitro* [25]. This further supports our results. Previous studies have shown that the lysyl oxidase (LOX) family plays an important role in stabilizing the extracellular matrix (ECM). In triple‐negative breast cancer, inhibition of LOX decreases extracellular matrix stability and increases drug penetration, which in turn induces apoptosis and resensitization to chemotherapy [26]. Similar studies have been conducted in ovarian [27], colon [28], and pancreatic cancers [29]. LOX is widely involved in the development of various tumors *in vivo* and is expected to be a potential intervention target. The CXCL12/CXCR4 axis may play an important role in the deproliferative response of pancreatic ductal adenocarcinoma, whereas loss of CXCR4 induces undifferentiated carcinomas with altered pancreatic phenotype without differentiated response [30]. Early studies found that C‐X‐C chemokine receptor type 4 (CXCR4) is overexpressed in many cancers, and that high expression of CXCR4 is always associated with early metastasis and poor prognosis [31]. CP is an oxidase involved in the regulation of systemic and intracellular iron levels [6]. However, the biological role of CP in ccRCC is rarely reported. In this study, we report that CP expression is upregulated in clinical ccRCC tissues using TCGA database data, and that high CP expression is strongly associated with more malignant clinical features and poor prognosis in ccRCC patients. To further verify the reliability of the results, we experimentally demonstrated that the mRNA and protein levels of CP in clear renal cell carcinoma were significantly higher than those of CP in normal renal tissue, and cellular experiments showed similar results. In order to further investigate the role of CP in the biology of clear renal cell carcinoma, both Matrigel method and wound healing assay demonstrated that the invasive ability of cells in the experimental group was significantly lower than that in the control group when the CP gene was downregulated. Previous studies have shown that CP can stabilize HIF‐1a and participate in its nuclear transport process, thereby regulating downstream VEGF expression [32]. Also, it was demonstrated that CP is a target gene of HIF‐1a. It is believed that hypoxia would promote the overexpression of HIF‐1a, thus forming a positive feedback loop and jointly promoting the biological deterioration of RCC [33]. Therefore, we hypothesize that high expression of CP may stabilize HIF‐1a protein, leading to the progression of RCC. However, the specific mechanism needs further study. Similar findings have been found in other diseases, where ceruloplasmin (CP) has been found to be one of the potential biomarkers for the diagnosis of hepatocellular carcinoma [34], CP can promote the growth and angiogenesis of tumor cells in breast cancer [35], plasma CP levels are significantly increased in patients with developing ovarian cancer, and the CP promoter shows significantly higher activity in ovarian cancer compared with normal organs [36, 37]. Combined with the above findings, bioinformatics analysis using mRNA microarray datasets from GEO and TCGA revealed some central genes and important pathways that may be associated with poor survival and metastasis in ccRCC. Meanwhile, CP expression level can be used as one of the lower survival indicators for assessing ccRCC patients, because it may be involved in the pathological process of distant metastasis of ccRCC.

In this study, our study has some shortcomings. First, the clinical sample size was too small and only four eligible frozen samples were analyzed by real‐time quantitative RT‐PCR and western blots. Therefore, in order to understand our results more accurately, we intend to collect more satisfactory samples later. Second, only one type of renal cancer cell line was validated, and the reliability was relatively low. In summary, in this study, based on the DEGs between ccRCC and normal tissues, DEGs were first identified by bioinformatics analysis, and then, potential key genes involved in ccRCC development were analyzed, and finally, central genes and signaling pathways that may be involved in ccRCC progression and metastasis were revealed. These epigenetic changes may be associated with poor overall survival in ccRCC. However, the specific biological roles of these candidate biomarkers in ccRCC and the specific molecular mechanisms involved in ccRCC metastasis require further investigation.

In summary, a total of 20 central genes were screened in this study; particularly, ALB, C3, LOX, HRG, CXCR4, GPC3, SLC12A3, CP, and CASR were closely related to the prognosis of RCC patients. In addition, high expression of CP was associated with lower overall survival in ccRCC patients. CP is expected to provide a theoretical basis for enriching ccRCC pathogenesis as well as new signaling pathways.

## Conflict of interest

The authors declare that they have no competing interests, and all authors should confirm its accuracy.

## Author contributions

BZ designed and directed the project. YZ, ZC, and JGC processed and analyzed the experimental data, and they had equal contributions to this research; YZ and XFC wrote and revised the manuscript. ZML conducted immunohistochemistry experiments. DHG conducted western blotting experiments. YDG and XFC conducted cell experiments. All authors have read and approved the manuscript and agreed to be responsible for all aspects of the research to ensure proper investigation and resolution of the accuracy or completeness of any part of the work.

## Data Availability

All data can be obtained by contacting the corresponding author.

## References

[1] Rini BI , Campbell SC and Escudier B (2009) Renal cell carcinoma. Lancet 373, 1119–1132. 19269025 10.1016/S0140-6736(09)60229-4

[2] Gupta K , Miller JD , Li JZ , Russell MW and Charbonneau C (2008) Epidemiologic and socioeconomic burden of metastatic renal cell carcinoma (mRCC): A literature review. Cancer Treat Rev 34, 193–205. 18313224 10.1016/j.ctrv.2007.12.001

[3] Crispen PL , Breau RH , Allmer C , Lohse CM , Cheville JC , Leibovich BC and Blute ML (2011) Lymph node dissection at the time of radical nephrectomy for high‐risk clear cell renal cell carcinoma: indications and recommendations for surgical templates. Eur Urol 59, 18–23. 20933322 10.1016/j.eururo.2010.08.042

[4] Capitanio U and Montorsi F (2016) Renal cancer. Lancet 387, 894–906. 26318520 10.1016/S0140-6736(15)00046-X

[5] Malati T (2007) Tumour markers: an overview. Indian J Clin Biochem Ijcb 22, 17. 23105677 10.1007/BF02913308 PMC3453798

[6] Osaki S , Johnson DA and Frieden E (1966) The possible significance of the ferrous oxidase activity of ceruloplasmin in normal human serum. J Biol Chem 241, 2746–2751. 5912351

[7] Daffada AAI and Young SP (1999) Coordinated regulation of ceruloplasmin and metallothionein mRNA by interleukin‐1 and copper in HepG2 cells. FEBS Lett 457, 214–218. 10471781 10.1016/s0014-5793(99)01036-4

[8] Persichini T , Maio N , di Patti MCB , Rizzo G , Colasanti M and Musci G (2010) Interleukin‐1β induces ceruloplasmin and ferroportin‐1 gene expression via MAP kinases and C/EBPβ, AP‐1, and NF‐κB activation. Neurosci Lett 484, 133–138. 20727382 10.1016/j.neulet.2010.08.034

[9] Raju KS , Alessandri G , Ziche M and Gullino PM (1982) Ceruloplasmin, copper ions, and angiogenesis. J Natl Cancer Inst 69, 1183–1188. 6182332

[10] Pejovic M , Djordjevic V , Ignjatovic I , Stamenic T and Stefanovic V (1997) Serum levels of some acute phase proteins in kidney and urinary tract urothelial cancers. Int Urol Nephrol 29, 427–432. 9405999 10.1007/BF02551108

[11] Doustjalali SR , Yusof R , Govindasamy GK , Bustam AZ , Pillay B and Hashim OH (2006) Patients with nasopharyngeal carcinoma demonstrate enhanced serum and tissue ceruloplasmin expression. J Med Invest 53, 20–28. 16537992 10.2152/jmi.53.20

[12] Bleu M , Gaulis S , Lopes R , Sprouffske K , Apfel V , Holwerda S , Pregnolato M , Yildiz U , Cordoʹ V , Dost AFM *et al*. (2019) PAX8 activates metabolic genes via enhancer elements in Renal Cell Carcinoma. Nat Commun 10, 3739. 31431624 10.1038/s41467-019-11672-1 PMC6702156

[13] Subramanian A , Tamayo P , Mootha VK , Mukherjee S , Ebert BL , Gillette MA , Paulovich A , Pomeroy SL , Golub TR , Lander ES *et al*. (2005) Gene set enrichment analysis: A knowledge‐based approach for interpreting genome‐wide expression profiles. Proc Natl Acad Sci USA 102, 15545–15550. 16199517 10.1073/pnas.0506580102 PMC1239896

[14] King HC and Sinha AA (2001) Gene expression profile analysis by DNA microarrays: promise and pitfalls. Jama J Am Med Assoc 286, 2280–2288. 10.1001/jama.286.18.2280 11710894

[15] Goka ET , Chaturvedi P , Lopez DTM and Lippman ME (2020) Rac signaling drives clear cell renal carcinoma tumor growth by priming the tumor microenvironment for an angiogenic switch. Mol Cancer Ther 19, 1462–1473. 32371578 10.1158/1535-7163.MCT-19-0762

[16] Escudier B , Eisen T , Stadler WM , Szczylik C , Oudard S , Siebels M , Negrier S , Chevreau C , Solska E , Desai AA *et al*. (2007) Sorafenib in advanced clear‐cell renal‐cell carcinoma. N Engl J Med 356, 125–134. 17215530 10.1056/NEJMoa060655

[17] Motzer RJ , Hutson TE , Tomczak P , Michaelson MD , Bukowski RM , Rixe O , Oudard S , Negrier S , Szczylik C , Kim ST *et al*. (2007) Sunitinib versus interferon alfa in metastatic renal‐cell carcinoma. N Engl J Med 356, 115–124. 17215529 10.1056/NEJMoa065044

[18] Mikami S , Mizuno R , Kosaka T , Saya H , Oya M and Okada Y (2015) Expression of TNF‐α and CD44 is implicated in poor prognosis, cancer cell invasion, metastasis and resistance to the sunitinib treatment in clear cell renal cell carcinomas. International Journal of Cancer 136, 1504–1514. 10.1002/ijc.29137 25123505

[19] Boguslawska J , Kedzierska H , Poplawski P , Rybicka B , Tanski Z and Piekielko‐Witkowska A (2016) Expression of genes involved in cellular adhesion and extracellular matrix remodeling correlates with poor survival of patients with renal cancer. J Urol 195, 1892–1902. 26631499 10.1016/j.juro.2015.11.050

[20] Wang X‐L , Hou L , Zhao C‐G , Tang Y , Zhang B , Zhao J‐Y and Wu Y‐B (2019) Screening of genes involved in epithelial‐mesenchymal transition and differential expression of complement‐related genes induced by PAX2 in renal tubules: screening of genes involved in EMT. Nephrology 24, 263–271. 29280536 10.1111/nep.13216 PMC6585862

[21] Guglietta S and Rescigno M (2016) Hypercoagulation and complement: connected players in tumor development and metastases. Semin Immunol 28, 578–586. 27876232 10.1016/j.smim.2016.10.011

[22] Guglietta S , Chiavelli A , Zagato E , Krieg C , Gandini S , Ravenda PS , Bazolli B , Lu B , Penna G and Rescigno M (2016) Coagulation induced by C3aR‐dependent NETosis drives protumorigenic neutrophils during small intestinal tumorigenesis. Nat Commun 7, 11037. 26996437 10.1038/ncomms11037 PMC4802169

[23] Um HJ , Min K , Kim DE and Kwon TK (2012) Withaferin A inhibits JAK/STAT3 signaling and induces apoptosis of human renal carcinoma Caki cells. Biochem Biophys Res Commun 427, 24–29. 22982675 10.1016/j.bbrc.2012.08.133

[24] Ueda K , Ogasawara N , Yonekura S , Matsunaga Y , Hoshino R , Kurose H , Chikui K , Uemura K , Nakiri M , Nishihara K *et al*. (2020) The prognostic value of systemic inflammatory markers in advanced renal cell carcinoma patients treated with molecular targeted therapies. Anticancer Res 40, 1739–1745. 32132082 10.21873/anticanres.14127

[25] Xu Y , Huang Y , Xu W , Zheng X , Yi X , Huang L , Wang Y and Wu K (2020) Activated hepatic stellate cells (HSCs) exert immunosuppressive effects in hepatocellular carcinoma by producing complement C3. OncoTargets Ther 13, 1497–1505. 10.2147/OTT.S234920 PMC7035898 32110047

[26] Saatci O , Kaymak A , Raza U , Ersan PG and Sahin O (2020) Targeting lysyl oxidase (LOX) overcomes chemotherapy resistance in triple negative breast cancer. Nat Commun 11, 2416. 32415208 10.1038/s41467-020-16199-4 PMC7229173

[27] Donato MD , Petrillo M , Martinelli E , Filippetti F and Gallo D (2017) Uncovering the role of nuclear Lysyl oxidase (LOX) in advanced high grade serous ovarian cancer. Gynecol Oncol 146, 170–178. 28495238 10.1016/j.ygyno.2017.05.001

[28] Shi XM , Zhao W , Yang YB and Lü BN (2017) Expression of LOX in colorectal cancer tissues and its relationship with progress and prognosis. Sichuan Xue Xue Bao Yi Xue Ban 48, 566–569. 29130678

[29] Ma W , Li T , Wu S , Xiuchao J , Wang A and Li H (2019) LOX and ACSL5 as potential relapse markers for pancreatic cancer patients. Cancer Biol Ther 20, 787–798. 30712446 10.1080/15384047.2018.1564565 PMC6605990

[30] Morita T , Kodama Y , Shiokawa M , Kuriyama K , Marui S , Kuwada T , Sogabe Y , Matsumori T , Kakiuchi N , Tomono T *et al*. (2020) CXCR4 in tumor epithelial cells mediates desmoplastic reaction in pancreatic ductal adenocarcinoma. Cancer Res 80, 4058–4070. 32606001 10.1158/0008-5472.CAN-19-2745

[31] Jäger B , Klatt D , Plappert L , Golpon H , Lienenklaus S , Barbosa PD , Schambach A and Prasse A (2020) CXCR4/MIF axis amplifies tumor growth and epithelial‐mesenchymal interaction in non‐small cell lung cancer. Cell Signal 73, 109672. 32428553 10.1016/j.cellsig.2020.109672

[32] Harned J , Ferrell J , Nagar S , Goralska M , Fleisher LN and McGahan MC (2012) Ceruloplasmin alters intracellular iron regulated proteins and pathways: Ferritin, transferrin receptor, glutamate and hypoxia‐inducible factor‐1α. Experimental Eye Research 97, 90–97. 10.1016/j.exer.2012.02.001 22343016 PMC3535017

[33] Martin F , Linden T , Katschinski DM , Oehme F , Flamme I , Mukhopadhyay CK , Eckhardt K , Tröger J , Barth S and Camenisch G . (2005) Copper‐dependent activation of hypoxia‐inducible factor (HIF)‐1: implications for ceruloplasmin regulation. Blood 105, 4613–4619. 15741220 10.1182/blood-2004-10-3980

[34] Nayak SB , Yashwanth S , Pinto SM , Bhat VR and Mayya SS (2005) Serum copper, ceruloplasmin, protein thiols and thiobarbituric acid reactive substance status in liver cancer associated with elevated levels of alpha‐fetoprotein. Indian J Physiol Pharmacol 49, 341–344. 16440854

[35] Pan Q , Kleer CG , Golen KLV , Irani J and Merajver SD (2002) Copper deficiency induced by tetrathiomolybdate suppresses tumor growth and angiogenesis. Cancer Res 62, 4854–4859. 12208730

[36] Nayak SB , Bhat VR and Mayya SS (2004) Serum copper, ceruloplasmin and thiobarbituric acid reactive substance status in patients with ovarian cancer. Indian J Physiol Pharmacol 48, 486. 15907060

[37] Lee CM , Lo HW , Shao R , Wang SC , Xia W , Gershenson DM and Hung MC (2004) Selective activation of ceruloplasmin promoter in ovarian tumors: potential use for gene therapy. Cancer Res 64, 1788. 14996741 10.1158/0008-5472.can-03-2551

